# Effect of returning home on university student hunger during South African COVID-19 lockdown

**DOI:** 10.1371/journal.pone.0340350

**Published:** 2026-01-09

**Authors:** Fezile Wagner, Unathi Kolanisi, Ryan G. Wagner, Lerato P. Makuapane, Mxolisi Masango, Francesc Xavier Gómez-Olivé

**Affiliations:** 1 Analytics and Institutional Research Unit (AIRU), University of the Witwatersrand, Johannesburg, South Africa; 2 Barrow Neurological Institute, Phoenix, Arizona, United States of America; 3 Department of Consumer Sciences, Faculty of Science and Agriculture, University of Zululand, KwaDlangezwa, South Africa; 4 MRC/Wits Rural Public Health and Health Transitions Research Unit (Agincourt), School of Public Health, Faculty of Health Sciences, University of the Witwatersrand, Johannesburg, South Africa; 5 Department of Demography and Population Studies, Faculty of Humanities, University of the Witwatersrand, Johannesburg, South Africa; 6 Institutional Planning Directorate (IPD), Cape Peninsula University of Technology, Cape Town, South Africa; Ain Shams University, EGYPT

## Abstract

The COVID-19 pandemic exacerbated hunger levels in South Africa, with an increase from 10% pre-pandemic to 23% during the pandemic. Pre-pandemic national and global research identified university students to be more vulnerable to hunger compared to the general population. This elevated risk is commonly associated with prevalent financial mismanagement in this group. However, research investigating the prevalence of hunger during the pandemic among this at-risk group is limited. This cross-sectional study aimed to assess the prevalence and determinants of hunger among students at a South African university during the COVID-19 lockdown, with particular focus on the effect of returning home. An online, self-administered survey produced a sample of 596 students. The Household Hunger Scale (HHS) was used to assess hunger. Most students (84%) who resided in on- or off-campus residences before the lockdown returned home during the lockdown. The weighted prevalence of hunger during lockdown was 16.4% (95%CI 13.6%, 19.6%). Bivariate analyses found living alone to be significantly associated with hunger, while multivariate analyses found that first-generation students (adjusted odds ratio (aOR) = 1.78; 95%CI: 1.04, 3.07, p = 0.015), financial aid recipients (aOR = 2.69; 95%CI: 1.47, 4.91, p = 0.001), and those experiencing financial stress/worry (aOR = 3.38; 95%CI: 1.85, 6.18, p < 0.001) were significantly more likely to experience hunger. The high prevalence of hunger during lockdown is concerning, the mandated return of students to their homes during the pandemic may have been a mitigating factor. Profiling students at risk for hunger may allow support services to implement targeted interventions when confronted with similar circumstances in future.

## Introduction

### Global and South African hunger context

Hunger is an enormous global challenge. The United Nations’ (UN) sustainable development goals (SDGs) have included the eradication of hunger by the year 2030 as Goal 2 [1]. Eliminating hunger and efforts towards attaining food security are crucial, as both are linked to lost productivity due to difficulties with concentration and links to malnutrition, which impede healthy development as well as increased susceptibility to illnesses [2]. South Africa, like other low- and middle-income countries (LMIC), faces persistent hunger challenges, primarily due to structural unemployment, inflation in basic food and fuel prices, and frequent power outages that disrupt food production and distribution systems [3].

Hunger and food insecurity are often conflated, but they refer to related yet distinct experiences [2]. According to the Food and Agriculture Organization (FAO) hunger is the physical sensation caused by insufficient food intake, while food insecurity refers to unsafe or limited nutritious food necessary for growth, development, and general healthy [2,4]. The United States Department of Agriculture (USDA) further distinguishes between *food insecurity without hunger*, which involves reduced diet quality or variety and *food insecurity with hunger*, which includes disrupted eating patterns, such as skipping meals, or going without food [5].

### Student hunger and food insecurity

Much previous work has examined university student food insecurity, while literature on university student hunger, is limited, especially in Africa [6–9]. Prior to the COVID-19 pandemic, studies reported that 66–84% of South African university students experienced food insecurity, while 5–60% reported going hungry due to insufficient access to food [6,9–11]. Financial mismanagement has also been identified as an important cause of food insecurity among university students, with some students prioritizing clothing and entertainment over food [12,13]. In addition, students often use financial aid to send remittances home, a practice prevalent in the African context [10]. South African literature has also raised concern, about the possible impact of the shift from catered to non-catered university residences, noting that while this approach reduces accommodation costs, it may unintentionally increase students’ risk of food insecurity and hunger [14].

### Study aim and rationale

Empirical research on the impact of the COVID-19 pandemic on student hunger has predominantly taken place in Western countries, with limited insights from LMICs, such as South Africa. To our knowledge, this is one of the first quantitative studies in South Africa, and among the first in Africa, to examine student hunger during the pandemic. By situating the study within the context of national lockdown measures, which included the mandatory return of students to their homes, we explore how these policy-driven changes in living arrangements may have influenced hunger outcomes. The current research therefore aims to assess the prevalence and determinants of hunger among students at a South African university during the COVID-19 lockdown, with particular focus on the impact of returning home.

### Impact of the COVID-19 lockdown

The COVID-19 pandemic had serious implications for both hunger and food insecurity in South Africa [15]. In response to the COVID-19 pandemic, the South African government declared a national state of disaster in March 2020 and implemented a five-level lockdown system, with Level 5 being the most restrictive. The pandemic and strict lockdown led to job loss which resulted in many households not having money to buy food [16–18]. Statistics suggest that the prevalence of hunger fluctuated between 16–23% in 2020, compared to around 10% in 2017 [19].

In 2020, the government implemented various targeted social protection measures to mitigate the impact of the pandemic and the associated economic disruptions on its citizens [20]. These measures were generally regarded as successful in reaching intended populations [21]. Temporary social protection included: increases in social protection measures such as child support grants and old age grants, a Temporary Employee/Employer Relief Scheme (TERS) which provided financial relief to employers and employees unable to work due to the operational downturn caused by the lockdown and other public health requirements [20,21]. Further to this, the government issued a special COVID-19 Social Relief of Distress (SRD) grant, whose stringent eligibility criteria included: not receiving any other social grant from the government, for students – not receiving financial aid, and not receiving any benefits from the unemployment insurance fund [20,22]. A means test validating applicants’ income through commercial banks formed part of the verification check [21]. Although the above may not have benefitted students directly, it may have contributed to improved household food security when pooled.

### Specific challenges for university students

Universities responded to the pandemic by shifting to remote learning and requiring students to vacate residences, encouraging them to return home. The challenges that students subsequently faced whilst learning remotely have been well documented [17,23]. These included poor access to devices and connectivity (data, bandwidth, and network reception) especially among students in more remote areas [24,25]. Some reported that their home environments were not conducive to studying, with many lacking dedicated study spaces, facing emotional distress, and facing financial stress as household situations worsened [17,23,25].

In terms of wellbeing, student hunger has been highlighted as an important psychosocial factor impacted by the pandemic [17,25,26]. Most research on this topic has emerged from the United States of America and has focused on food insecurity, with mixed findings showing both increases and decreases in its prevalence [13,27,28]. However, the most consistent finding is that returning home often improved students’ food security status [13,27,28].

Despite these findings, there is a dearth of literature on how the COVID-19 pandemic and lockdown impacted the hunger status of university students in South Africa. Understanding this impact is important, as food insecurity and hunger have been linked with poor academic performance, negative health outcomes as well as an increased likelihood of risky sexual behaviour [9,29].

## Methods

### Sample

The study was carried out at an urban university in Johannesburg, South Africa with ~41,000 students enrolled in 2020. Although the university is located in an urban area, its student body is drawn from a mixed catchment, including both rural and urban areas across South Africa. Sixty percent of these students were pursuing undergraduate studies, with 55% of them female and the majority (61%) being Black Africans. The university has a diverse student body representing all races and all South African official languages.

The current article analyses a sub-sample of a larger study, whose overall aim was to understand the impact of COVID-19 on the university community [23]. For the present analysis, the sampling frame comprised all first-time, first-year students registered full-time for an undergraduate degree in 2020 (approximately 5,700 students). And the inclusion criteria required that students be 18 years or older at the time of the survey. Only students meeting these inclusion criteria were included in the analysis.

Whilst the entire population was invited to participate in the study, the minimum sample required to power the study sufficiently was 434. This was determined using Cochran’s equation, corrected for finite populations [30].

Expressed as an equation:


$$n_{0\;=\;\frac{Z^{\text{2}}p(\text{1}\;-p)}{e^{\text{2}}}\;\;\;\text{and}\;\;\;n=\;\frac{n_{0}}{\text{1}\;+\;\frac{n_{0\;-\text{1}}}{N}}}$$

Where:

n: Required sample size.

Z: z-score corresponding to the confidence level.

p: Estimated proportion of the population.

e: Margin of error.

n: Corrected sample size for a finite population.

n₀: Initial sample size calculated using Cochran’s formula.

N: Total population size.

The assumptions included a population size 5 700, 99% level of confidence, 5% of marginal error, and a hunger prevalence of 23%. The final sample size also met adequacy criteria for logistic regression, exceeding the conventional threshold of at least 10 events per variable.

### Questionnaire development and validation

The questionnaire used in this study formed part of a broader institutional survey on the impact of COVID-19 on the university community and was developed by a multidisciplinary team including epidemiologists, psychologists, and higher education researchers [23]. The instrument combined standardised validated measures with study-specific items informed by prior institutional surveys, evidence on South African students’ and households’ experiences during the lockdown [17,23–25], and national and international studies documenting the socioeconomic impacts of COVID-19 [2,15,16,18,19].

Hunger was measured using the three-item Household Hunger Scale (HHS), a validated tool widely employed in South Africa and other LMICs [31,32]. Given the established psychometric properties of the HHS, including strong cross-cultural validity, no additional test–retest reliability or measurement invariance analyses were undertaken in this study.

Draft items were reviewed by content experts for face and content validity, and the full survey was piloted with a small group of first-year students to assess clarity, comprehensibility, and completion time, which resulted in minor wording adjustments. The questionnaire was administered in English, the primary language of instruction at the university; while formal translation was not required, items were phrased in simple, accessible language to support students for whom English is an additional language.

### Data collection

Using a cross-sectional research design, data collection took place between September and October 2020, during South Africa’s COVID-19 lockdown levels Two and One, which represented the post-peak phase of the national lockdown. Following ethical clearance, a list of student email addresses meeting the inclusion criteria was extracted from the university’s data warehouse. As part of the larger institutional study, all eligible first-time, first-year students were invited to participate in an online survey. The recruitment process for the study was initiated by sending emails to all students at the University who fulfilled the inclusion criteria. Following acceptance via an online consent process, participants completed a self-administered online survey, using the Research Electronic Data Capture (REDCap) platform [33]. It is important to acknowledge that students without stable internet access may be underrepresented in the sample. An automated follow-up reminder email was sent to students who had not participated within the first week. No multistage cluster sampling was applied; all data were collected from a single institution using the invitation procedure described above.

## Measures

### Household hunger

The study employed the three-item Household Hunger Scale (HHS) to assess household hunger [31]. The HHS is a tool commonly used in South Africa to assess household hunger and has been validated for cross-cultural use [32]. The three-item HHS assesses whether, in the past four weeks, there was no food in the household, anyone went to bed hungry, or anyone went a whole day without eating due to lack of food. The scale uses a four-week recall and is adaptable to various settings allowing for applicability to different contexts. Participants respond to a four-point Likert scale for each item – ranging from 0 (never) to 3 (often – more than 10 times in the last 4 weeks). Following the recoding of data in accordance with the HHS guidelines [31], the summed responses are then used to determine the level of hunger (a score of 0–1 indicating little to no hunger; 2–3 indicating moderate hunger; and 4–6 indicating severe hunger). These parameters have been used previously in similar studies [9,34]. The current study defined ‘hunger’, the outcome of interest in the analyses, as those scoring 2–6, and defined ‘no hunger’ as those with a score of 0–1. This tool exhibited good internal consistency in the current study, with Cronbach α value of 0.92.

### Socio-demographic characteristics

Socio-demographic characteristics examined in the study included sex (‘male’ or ‘female’), race (‘Black African’, ‘White’, ‘Coloured/mixed race’, ‘Indian and Chinese’), first-generation status (‘yes’ or ‘no’) denoting students who were first in their family to attend university, discipline of study (‘Commerce, Law and Management’, ‘Engineering’, ‘Health Sciences’, ‘Humanities’, and ‘Sciences’), financial aid status (coded as ‘yes’, for those who received funding, or ‘no’ for those who did not receive funding). Age was treated as a continuous variable in descriptive statistics, where the mean and standard deviation were used to summarise central tendency and variability across the sample. For the logistic regression, however, age was categorized into four groups (18, 19, 20, and >20 years). The categories were selected to reflect typical first-year entry ages (18–20), with the > 20 year age group capturing older or non-traditional entrants. Linearity with the log odds of hunger was assessed before creating these categories. Indian and Chinese students were grouped due to the small number of Chinese students in the sample which made separate statistical analysis unfeasible. Finally, high schools were classified by quintile (1–5 and ‘other’ denoting those who attended private or international high schools or those whose high schools were unknown). The school quintile is used in South Africa to classify public schools based on the socio-economic conditions of the communities they serve. Quintile 1 schools are found in low resource areas, while quintile 5 schools are found in the most affluent communities [35]. The high school quintile classifications were grouped into three in the multivariate analysis: ‘Quintile 1-3’ and ‘Quintile 4 & 5’ and ‘Other’. This categorization aligns with the South African Department of Basic Education’s classification system and distinguishes schools serving low-income communities from those serving more affluent ones.

### COVID-19 lockdown factors

Factors arising from the pandemic were considered. The variable ‘Place of residence during lockdown’ reflected where the students stayed during lockdown and was coded as ‘on- or off-campus residence’, ‘with relatives’, ‘at home’ and ‘no stable accommodation’. COVID-19 exposure was captured through the variable ‘Self-reported COVID-19 infection (of participant and/or close friends and family)’, based on a single yes/no item asking whether the respondent or someone close to them had tested positive by the time of the survey. The number of people currently living in the household was also captured and coded as ‘Living alone’, ‘2’, ‘3-4’ and ‘5 or more’. Other covariates included where the household was situated during lockdown (coded as ‘City/ Suburb’, ‘Township’, ‘Town’ and ‘Village/farm’); whether there was financial stress/ worry during lockdown (coded as ‘Yes’ or ‘No’); and finally the variable ‘Limited workspace at home’ to capture whether the student had a quiet and dedicated area conducive to studying and attending remote classes. This was self-reported and coded as ‘Yes’ if the student indicated workspace constraints and ‘No’ if adequate workspace was available.

### South Africa’s lockdown context

In response to the COVID-19 pandemic, the South African government instituted a national state of disaster on 15 March 2020 [36]. The government then introduced a lockdown, guided by 5 alert levels [37]. Lockdown restrictions were applied at a national level. Alert level 5 [38] was the most stringent level, confining people to places of residence, with alert level 1 having more relaxed restrictions including a curfew between 00h00 and 04h00 daily [39]. Universities adopted a new learning mode, allowing the academic programme to continue remotely [25,40,41]. Groups of students were reintroduced into campus as the lockdown levels eased [40–42]. During the lockdown, ‘essential services’ including the operation of grocery stores and corner/convenience shops were not restricted [38]. Similarly, the transportation and logistics of essential goods, such as food, faced no limitations [38]. Commuter transport services were allowed to operate, albeit with capacity restrictions, to facilitate the movement of individuals involved in rendering or obtaining essential goods or services [38]. Moreover, the acquisition of essential goods, like food, was explicitly permitted [38]. These guidelines were relevant country wide, including in rural and urban settings.

### Analytic approach

Data were cleaned and analysed using STATA (version 17; College Station, Texas, USA). Descriptive analyses were performed for all variables, with proportions and 95% confidence intervals (95% CI) reported when appropriate. The Pearson’s chi-square test was used to compare categorical variables with hunger, and the Mann-Whitney U test was used to compare the continuous variable (age).

Data were weighted using post-stratification weighting based on joint distribution (sex and race) from institutional administrative records to adjust for the differences between the students who participated in the study and the underlying student population. These weights were applied to all descriptive and regression analyses. Missing data were handled using listwise deletion, resulting in the analytic sample of 596 students described above. Variables included in the logistic regression model were selected using a forward stepwise regression, with a cut-off inclusion of p-values ≤ 0.20 for the model and adjusted Odds Ratios (aOR) with confidence intervals were reported. Significance was defined at p-values <0.05. To assess model diagnostics, we tested for multicollinearity using the Variance Inflation Factor (VIF). Model fit was assessed using the Hosmer–Lemeshow goodness-of-fit test, and explanatory power was evaluated using McFadden’s pseudo R².

### Ethical statement

Ethical approval was received from the University of the Witwatersrand Human Research Ethics Committee (Non-medical) (H20/06/22). Written permission was received from the Office of the university Registrar to conduct the study. Each participant provided digital consent before participation.

## Results

Of the 5,684 students who met the inclusion criteria and were invited to participate in this study, 726 (12.8%) agreed to participate; 130 were excluded from the analyses due to missing data in key variables including: hunger status, school quintile, and place of residence, yielding a final analytical sample of 596 (10.5%) participants, representing 17% of the main study sample. The weighted prevalence of hunger among participants was 16.4% (95% CI 13.6, 19.6).

The results are presented in three parts. First, we describe the socio-demographic profile of the study sample and examine variations in hunger prevalence across key background characteristics. Second, we explore associations between hunger and contextual factors related to the COVID-19 lockdown, including changes in living arrangements, household circumstances, and learning environments. Finally, we present findings from a multivariate logistic regression to identify the strongest independent predictors of hunger after adjusting for potential confounders.

To understand which socio-demographic groups were most affected by hunger, we examined hunger prevalence across key background variables, including race, age, high school quintile, and financial aid status (Table 1). Statistically significant differences were observed for several of these factors. In terms of hunger levels by race (p < 0.001), Black African students experienced substantially higher hunger levels than their White, Indian, and Coloured counterparts. A significant age gradient (p = 0.003) was observed with younger students reporting lower hunger levels than older participants. Students who had attended lower quintile high schools were significantly more likely to experience hunger than those from quintile 5 or private schools (p < 0.001), suggesting persistent educational and economic disadvantage. First-generation university students had nearly three times the hunger prevalence of non-first-generation students (p < 0.001), while students receiving financial aid were almost four times more likely to report hunger than those who were not (p < 0.001). Together, these findings suggest strong structural and intergenerational links to food insecurity within the student population.

**Table 1:** Weighted socio-demographic characteristics of students by hunger status.

Variable	Sample %	No hunger %	Hunger %	p-value
**Total hunger prevalence**	–	83.6%	16.4%	–
**Sex**				
Female	57.0%	85.5%	14.5%	0.124
Male	43.0%	81.1%	18.9%	
**Race** ^1^				
Black African	69.0%	77.1%	22.8%	<0.001*
Coloured/mixed race	4.6%	97.5%	2.6%	
Indian	12.8%	95.9%	4.1%	
White	13.6%	100.0%	0.0%	
**Age [M (SD)]** ^2^	20.0 (± 2.3)	19.8 (± 2.0)	20.1 (± 3.3)	0.003*
**High school quintile**				
Quintile 1	6.6%	55.0%	45.0%	<0.001*
Quintile 2	10.8%	62.1%	37.9%	
Quintile 3	14.1%	77.7%	22.3%	
Quintile 4	11.6%	82.4%	17.6%	
Quintile 5	33.7%	88.7%	11.3%	
Private/ Int./ Other	23.2%	98.6%	1.4%	
**Discipline of study**				
Commerce, Law & Management	14.3%	84.3%	15.7%	0.466
Engineering	23.3%	81.5%	18.5%	
Health Sciences	13.4%	90.0%	10.0%	
Humanities	30.8%	81.3%	18.7%	
Sciences	18.3%	85.0%	15.0%	
**First-generation status**				
First-generation	43.0%	72.6%	27.4%	<0.001*
Non-first-generation	57.0%	91.9%	8.1%	
**Financial-aid recipient**				
Yes	50.1%	73.7%	26.3%	<0.001*
No	49.5%	93.3%	6.7%	
**Self- reported disability status**				
Yes	3.0%	92.6%	7.4%	0.277
No	97.0%	83.3%	16.7%	

* Significant at p < 0.05 (p-values represent frequency diﬀerences between no hunger and hunger); Data weighted by sex and race.

^1^Race in South Africa is closely linked to socio-economic disparities, with different racial groups often having varying access to resources like education, healthcare, and employment [43].

^2^Age described by median values, standard deviation, and the Mann-Whitney U test.

We next explored how experiences during the COVID-19 lockdown, such as changes in residence, financial strain, and home learning environments, were associated with hunger (Table 2). Several contextual factors, including living alone, household location, financial stress, and lack of workspace at home, were significantly associated with higher hunger prevalence. Most study participants (84.0%) who were residing in on- or off-campus residence prior to the lockdown returned home. In total, 87.8% reported residing at home during the lockdown. The bivariate analysis indicated a correlation between hunger status and place of residence, with having no stable accommodation being significantly associated with increased levels of hunger (p < 0.014), however this group represented a small proportion and results should be interpreted cautiously. Living in larger households (three or more members) and living in cities and suburbs were significantly associated with decreased levels of hunger (p < 0.001). Financial stress during lockdown emerged as a key driver of hunger, with 24.5% of financially stressed students reporting hunger compared to just 6.4% of those not experiencing financial worry (p < 0.001). Similarly, students having limited workspace at home were more than twice as likely to report hunger as those with adequate study environments (p < 0.001).

**Table 2:** Weighted COVID-19 lockdown factors associated with hunger.

Variable	sample %	No hunger %	Hunger %	p-value
**Place of residence during lockdown**				
On or off-campus residence	5.1%	83.4%	16.7%	0.014*
With Relatives	6.8%	81.5%	18.5%	
At home	87.8%	84.2%	15.9%	
No stable accommodation	0.4%	0.0%	100.0%	
**Self- reported COVID-19 infection (of participant and/or close friends and family)**				
Yes	7.2%	84.5%	15.5%	0.781
No	92.8%	83.5%	16.5%	
**Household size**				
Living alone	7.4%	69.0%	31.0%	<0.001*
2	18.5%	72.2%	27.8%	
3-4	38.1%	93.6%	6.4%	
5 or more	36.0%	81.9%	18.1%	
**Household location during lockdown situated in:**				
City/ Suburb	55.7%	89.2%	10.8%	<0.001*
Town	7.0%	86.3%	13.7%	
Township	23.1%	76.3%	23.7%	
Village/farm	14.2%	71.9%	28.1%	
**Financial stress/ worry**				
Yes	55.1%	75.5%	24.5%	<0.001*
No	44.9%	93.6%	6.4%	
**Limited workspace at home**				
Yes	54.4%	77.4%	22.6%	<0.001*
No	45.6%	91.0%	9.0%	

* Significant at p < 0.05 (p-values represent frequency diﬀerences between no hunger and hunger); Data presented as weighted values weighted for sex and race.

Multivariate logistic regression was conducted to identify which factors remained significantly associated with hunger after adjusting for other variables (Table 3). The model demonstrated good calibration (Hosmer–Lemeshow χ²(8) = 7.49, p = 0.485) and explained a modest proportion of the variance in the outcome (McFadden’s pseudo R² = 0.24). In addition, variables in the final model had VIF values below 5, indicating no problematic multicollinearity. Variables that were significant in the bivariate analysis but not retained in the final model, such as household size and location, did not meet the inclusion criterion (p ≤ 0.20).

**Table 3:** Multivariate logistic regression predicting socio-demographic and lockdown related correlates to hunger.

	aOR (95 CI)	p-value
**Gender (reference: Female)**		
Male	0.79 (0.47, 1.34)	0.385
**Age group in years (reference:18)**		
19	0.35 (0.10, 1.24)	0.103
20	0.54 (0.14, 2.00)	0.353
> 20	1.03 (0.28, 3.82)	0.967
**High school quintile category**^1^ **(reference: Quintiles 1–3)**		
Quintiles 4&5	0.47 (0.27, 0.80)	0.005*
Other	0.08 (0.02, 0.38)	0.001*
**First-generation status (reference: No)**		
Yes	1.78 (1.04, 3.07)	0.037*
**Financial-aid recipient (reference: No)**		
Yes	2.69 (1.47, 4.91)	0.001*
**Self- reported COVID-19 infection (of participant and/or close friends and family) (reference: No)**		
Yes	2.00 (0.72, 5.54)	0.208
**Financial stress/ worry (reference: No)**		
Yes	3.38 (1.85, 6.18)	<0.001*
**Limited workspace at home (reference: No)**		
Yes	1.57 (0.90, 2.73)	0.113

* Significant at p < 0.05.

^1^Percentages for high school quintile categories: Quintiles 1–3 = 31.5%, Quintiles 4–5 = 45.3%, Other = 23.2%.

Being a first-generation student almost doubled the odds of hunger (aOR = 1.78; 95% CI: 1.04, 3.07, p = 0.037) and receiving financial aid nearly tripled the odds of experiencing hunger (aOR = 2.69; 95% CI: 1.47, 4.91, p = 0.001). These findings indicate that both familial educational background and economic need contribute significantly to hunger risk, even after adjusting for other variables. Furthermore, financial stress/worry increased the odds of hunger more than three-fold (aOR = 3.38; 95% CI: 1.85, 6.18, p < 0.001), reinforcing the importance of psychosocial and economic factors. In contrast, the multivariate logistic regression showed that participants who attended more affluent high schools as well as those falling in the ‘other’ high school category were significantly less likely to experience hunger (aOR = 0.47 and 0.08 respectively), suggesting that earlier educational advantage may have lasting protective effects. Other variables such as gender, age, and limited workspace were not independently associated with hunger in the adjusted model.

## Discussion

This study addresses a significant gap in the quantitative literature on student hunger during the COVID-19 pandemic in South Africa. In qualitative studies, hunger has been cited by South African adolescents, including University students, as one of the biggest challenges brought on by the COVID-19 pandemic [17]. Yet quantitative evidence of this, especially in LMICs, including South Africa, is limited. Our findings therefore provide the population-based estimates of student hunger during the pandemic and allow comparison with both pre-pandemic student data and national household estimates.

This research found the weighted prevalence of hunger among university students to be 16.4% (95% CI 13.6%, 19.6%) using a tool with a four-week recall period. This estimate is ~ 7% lower than the pre-pandemic hunger prevalence reported at the same university using the same measure and methodology [9] and sits within the wider range of hunger and food insecurity estimates reported among students at other South African universities (5–60%) [6,9–11,14]. Compared with national data, where household hunger fluctuated between 16–23% during 2020 following an increase from around 10% in 2017 [15,19], our student estimate is similar to the lower end of the national range, suggesting that, while students remained vulnerable, their hunger levels were not necessarily higher than those of the general population during this period. The study also highlighted several factors that were associated with increased levels of hunger including being a first-generation student, a recipient of financial aid, and experiencing stress or worry due to finances. Those attending well-resourced, private, or international high schools were significantly less likely to experience hunger.

At the time of the current survey, most students (87.8%) were living at home, having returned from on- or off-campus residences due to lockdown regulations. This shift in living arrangements likely contributed to lower reported hunger levels. Studies conducted in South Africa prior to the pandemic suggest that students who lived with their parents had the lowest risk of hunger, and our findings are consistent with this trend [10]. International evidence from the United States similarly indicates that students who returned home during the COVID-19 pandemic often experienced improved food security relative to those who remained independent [13,27,28], suggesting that pooled household resources and access to family support may have buffered some students from the worst effects of the crisis.

The home environment likely supported students through improved access to meals, household food sharing, and caregiving by family members, particularly women, who are often responsible for obtaining and preparing food [44]. It is crucial to recognise the potential influence of the various social protection measures implemented by the South African government during this time [20,22]. However, given that social grants are means tested, only a limited number of the students under study would likely have qualified (when using high school quintile as a proxy for socio-economic status). Any benefits from these measures would therefore have been indirect, for example, through increased household income for family members who did qualify.

Although factors related to lockdown, such as changes in household size and location, were statistically significant in the bivariate analysis (p < 0.001), they did not meet the inclusion criterion (p ≤ 0.20) for the multivariate model and were therefore not retained. This may be because, once other covariates were included, their independent association with hunger was reduced, possibly due to shared variance with related factors such as income, employment status, or place of residence. Students who lived alone, experienced the highest levels of hunger (31.0%), reinforcing earlier findings that students who live alone are especially vulnerable to food insecurity and hunger [6,11]. Similarly, students who resided in villages or farms reported the highest prevalence of hunger (28.1%), consistent with South African evidence showing that households in traditional rural areas were more likely to run out of money for food and experience hunger during the COVID-19 pandemic [16,18].

The current research reaffirms the importance of socio-economic factors in shaping hunger risk. First-generation students were significantly more likely to report hunger, consistent with research showing that limited parental education and employment reduce access to food [10,45,46]. The increased odds of hunger among students receiving financial aid may appear counterintuitive, however, it mirrors previous findings suggesting low-income students remain financially strained even after aid [10,11,27,46]. Delays in financial aid disbursement during the pandemic further compounded food insecurity and hunger among university students in South Africa [17]. Financial stress emerged as a strong predictor, echoing similar studies linking perceived financial strain to hunger and broader literature on the psychosocial dimensions of food insecurity among young people [34]. Finally, students matriculating from high schools located in affluent communities were less likely to experience hunger, likely due to access to economic resources during the crisis [47] and the persistence of structural inequalities in South Africa [43].

To our knowledge, this is one of the first studies from South Africa and Africa to assess the prevalence and predictors of student hunger during the COVID-19 pandemic. Our response rate (10.5%) was higher than comparable surveys (range 4.4–6.9%) [13,27,28]. Moreover, the sample was adequately powered for statistical analysis. However, the following limitations should be considered when interpreting the results:

i) The cross-sectional design limits causal inference regarding the pandemic’s impact on hunger, and the findings reflect conditions during the specific period of data collection (September–October 2020), which may differ from other stages of the pandemic.
ii) Although the finding indicating an association between a lack of stable accommodation and hunger provides valuable insight, the small number of students without stable accommodation may limit the interpretation.
iii) Generalizability is limited, as the work was carried out at a single University. However, the student body of the university is diverse and broadly reflective of the multicultural nature of the South African higher education sector.
iv) The absence of household-level data limited the ability to control for family background in regression models.
v) Non-response bias related to the response rate, as well as self-selection and self-report bias are potential limitations, particularly for subjective measures such as workspace adequacy and financial stress. Data were weighted by sex and race to partially mitigate potential differences between the sample and the underlying student population.
vi) Hunger may be underreported due to stigma or social desirability bias, particularly in a university setting where students may feel uncomfortable disclosing. Such underreporting could result in an underestimation of the true prevalence.
vii) Finally, although we used a widely validated hunger measure that demonstrated excellent internal consistency in this sample, we did not conduct additional psychometric analyses such as test–retest reliability or measurement invariance; future work could explore these aspects in more detail.

## Conclusion

This is one of the first quantitative studies from South Africa to use a validated tool to assess the prevalence and predictors of hunger among undergraduate students during the COVID-19 pandemic. Nearly one-fifth (16.4%) of undergraduate students reported hunger. Although hunger levels were lower than in pre-pandemic studies, our findings support international evidence that returning home may have buffered some students from hunger.

Yet, hunger remains a pressing concern, especially for first-generation students, reliant on financial aid, or experiencing financial stress. These groups are disproportionately affected by structural inequities in higher education and should be considered high-risk and prioritized for institutional support. In practical terms, universities could incorporate brief hunger screening tools into routine academic advising, financial aid, or wellness sessions, and develop targeted support packages (e.g., emergency food assistance, meal vouchers, financial counselling) for students identified as at risk.

Design of social protection and student funding schemes should take into account the specific vulnerabilities of university students, including those who support households through remittances. Future research should examine how these structural inequalities interact with student support systems to shape long-term educational and health outcomes, ideally through longitudinal and mixed-methods designs that can capture both trajectories and lived experiences.
